# Supplementary material for: Cost analysis of chronic pain due to musculoskeletal disorders in Chile
Source: PLoS One. 2022 Oct 27;17(10):e0273667. doi: 10.1371/journal.pone.0273667 (PMC9612497; doi:10.1371/journal.pone.0273667)
Supplement: Attachment — Submitted filename: Revision notes for manuscript.docx [file pone.0273667.s001.docx]

Dear Editor,

After making a careful review of our manuscript, based on the suggestions made by the reviewers, we have proceeded to send a new version of the manuscript “**Cost analysis of chronic pain due to musculoskeletal disorders in Chile**”. You will find in the system a new consolidated manuscript and the document with track changes. We thank the reviewers for all the comments that certainly helped us to enhance our manuscript.

Please find in revision notes document the answers to each comment made by the reviewers and to the manuscript.

Kind Regards,

**Dr. Manuel Espinoza, MD MSc PhD**

On behalf of the authors

^1^ Associate Professor, Department of Public Health, Pontificia Universidad Católica de Chile, Diagonal Paraguay 362, Piso 2, Santiago, Chile.

Email: manuel.espinoza@uc.cl

**Revision notes for manuscript: Cost analysis of chronic pain due to musculoskeletal disorders in Chile**

Reviewer #1: The authors aim to estimate the direct and indirect economic burden of chronic pain in Chile focusing on 5 diseases with LBA and FMS contributing to the highest cost borne for chronic pain amongst MsKDs.

| Reviewer comment | Response |
| --- | --- |
| The entire manuscript is wordy and has language errors. Writing needs to be improved to add clarity to the manuscript. | Thanks for your comment, we revised the text and made the necessary changes. |
| The methodology is confusing. Type of study, subheadings, number of patients assessed etc need to be mentioned. A flow chart can be added for better clarity | Many thanks, we have edited the text for a better clarity. The type of study is a cost-analysis study based on mathematical decision model. We have provided subheadings |
| The abbreviations in the tables need expansion. A common way of writing should be followed ($ mentioned in the upper part of table 2 only) | Thanks, we have provided a foot note with the abbreviation’s meanings.  We have corrected the $ in table 2. |

Reviewer #2: The manuscript represents the expected cost, i.e., total cost, sick leave payment and productivity losses associated with chronic pain and related musculoskeletal diseases in the Chilean adult population. Apart from total therapeutic cost, the study has shown the impact of pain management with respect to social security and productivity losses concerning human capital. Following are the comments that could be considered for improving the manuscript:
Major comments:

| **Reviewer comment** | **Response** |
| --- | --- |
| 1. The perspective of cost analysis could be explained more explicitly. | Thanks, we added some text in the introduction |
| 2. Justification shall be given in adopting a time horizon of 1 year as the musculoskeletal disorders are progressive in nature and last for a lifetime | Thanks for your comment, we have provided a justification. |
| 3. Although the model was adapted from the previous study, the present manuscript should include a schematic representation of the Markov model. | Good comment. We have provided a figure that represents the Markov model. |
| 4. Does the model account for drug-induced side effects, particularly when NSAIDs, opioid analgesics, and oral corticosteroids are used to treat chronic refractory pain? Because failure could lead to underestimation of cost. | Thanks for this comment, Yes they are included and we have provided in the text the items that the clinical management included. |
| 5. Whether are the costs or resources incurred for invasive management of patients with server chronic pain included while estimating the total therapeutic cost? | Yes, they are included |
| 6. The level of utilization has a significant impact on unit cost estimates. Please explain how COVID-19 management affects the utilization of pain management services in Chile and how this was taken into account when evaluating unit cost for the current study. | The data used for cost estimation was pre-pandemic, thus it has no influence in the informed costs. |
| 7. Does the statement in lines 299–301 constitute limitation in evaluating the cost of sick leaves? | Thanks for this comment, I do limits the cost to those with a formal work contract |

Minor comments:

| 8. Can the decimal points in table 1 be replaced with a dot instead of a comma? | Thanks, we have changed for dots. |
| --- | --- |
| 9. The use of upper and lower case in table 1 is inconsistent. | Thanks, we have made the corrections |
| 10. Sentences in the following lines need to be rephrased or corrected: Lines 302–303; line 74. | Thanks, we have edited the text. |
