# Supplementary material for: Cost analysis of chronic pain due to musculoskeletal disorders in Chile
Source: PLoS One. 2022 Oct 27;17(10):e0273667. doi: 10.1371/journal.pone.0273667 (PMC9612497; doi:10.1371/journal.pone.0273667)
Supplement: Attachment — Submitted filename: Revision notes for manuscript 12.8.22.docx [file pone.0273667.s002.docx]

Dear Editor,

Thanks for the review of our manuscript. We have proceeded to send a new version of the manuscript “**Cost analysis of chronic pain due to musculoskeletal disorders in Chile**” including the comments of the reviewer. You will find in the system a new consolidated manuscript and the document with track changes. We thank the reviewers for all the comments that certainly helped us to enhance our manuscript.

Please find in revision notes document the answers the comment made by the reviewer.

Kind Regards,

**Dr. Manuel Espinoza, MD MSc PhD**

On behalf of the authors

^1^ Associate Professor, Department of Public Health, Pontificia Universidad Católica de Chile, Diagonal Paraguay 362, Piso 2, Santiago, Chile.

Email: manuel.espinoza@uc.cl

**Revision notes for manuscript: Cost analysis of chronic pain due to musculoskeletal disorders in Chile**

Reviewer #1:

| Reviewer comment | Response |
| --- | --- |
| Thank you for making the revisions. The manuscript looks much better now. Kindly expand the abbreviations in Table 1. | Thanks for your comment, we have replaced the abbreviations for the full text on table 1. |
